# Applications and Future Prospects of Plant Multi-Omics Technologies in Precision Agriculture

**DOI:** 10.3390/ijms27209034

**Published:** 2026-10-12

**Authors:** Tao Tong, Qingxia Wang, Fanrong Zeng, Shuzhen Ye, Zhijuan Ji, Zhonghua Chen, Yanli Wang, Younan Ouyang

**Affiliations:** 1 State Key Laboratory for Rice Biology and Breeding, China National Rice Research Institute, Hangzhou 311401, China; 2 Research and Development Center of Rice Cropping Technology, China National Rice Research Institute, Hangzhou 311401, China; 3 MARA Key Laboratory of Sustainable Crop Production in the Middle Reaches of the Yangtze River (Co-Construction by Ministry and Province), College of Agriculture, Yangtze University, Jingzhou 434025, China; 4 School of Agriculture, Food and Wine, Adelaide University, Glen Osmond, SA 5064, Australia; 5 State Key Laboratory for Quality and Safety of Agro-Products, Key Laboratory of Agricultural Microbiome of Zhejiang Province, Key Laboratory of Biotechnology in Plant Protection of MARA, Institute of Plant Protection and Microbiology, Zhejiang Academy of Agricultural Sciences, Hangzhou 310021, China

**Keywords:** crops, multi-omics, agricultural production, sustainable agriculture

## Abstract

Against the backdrop of global population growth, intensifying climate change, and limited arable land, ensuring food security and promoting sustainable agriculture have become critical issues. The limitations of conventional agricultural systems are becoming increasingly apparent, creating an urgent need for innovation driven by science and technology. In this context, plant multi-omics technologies serve as the foundational data and technical cornerstone of systems biology and precision agriculture. Integrating multi-level data from genomics, transcriptomics, proteomics, metabolomics, epigenomics, and microbiomics offers substantial advantages for systematically understanding and improving plants. Unlike previous reviews that mainly catalog omics technologies or focus on individual crops, this review adopts a unified discovery-to-translation framework and critically evaluates the strength of evidence across applications. This review focuses on cutting-edge applications of multi-omics in three key areas: improving the traits of cereal crops (rice, wheat and maize), remediating soil health, and innovating products such as biofertilizers and biostimulants for agriculture. We systematically explore the roles of multi-omics in accelerating breeding processes, deciphering complex agronomic traits, restoring soil ecosystems, and constructing intelligent decision-support systems. The review also examines current challenges, including the complexity of data integration and the difficulties of predicting genotype-by-environment interactions. Furthermore, it outlines future directions involving deeper convergence with artificial intelligence, high-throughput phenomics, and digital twins. In summary, plant multi-omics technology is a vital tool for unravelling the mysteries of plant life and is also a key enabler of intelligent, precise and sustainable agricultural transformation.

## 1. Introduction

Agriculture is the cornerstone for the development of human society. However, the global agricultural system is currently under multiple pressures from population growth, climate change, and environmental pollution, as well as declining food production sustainability [1]. The Food and Agriculture Organization (FAO) predicts that the global population will exceed 9.7 billion by 2050, with food demand expected to increase by between 35% and 56% [2,3]. This will place substantial demands on agricultural production [4]. Meanwhile, climate change leads to frequent extreme weather events such as droughts, floods, and heatwaves, which threaten stable crop productivity and yield potential [5,6]. Furthermore, long-term intensive monoculture and excessive use of chemical fertilizers and pesticides have exacerbated soil degradation, biodiversity loss, and environmental pollution, thereby undermining the sustainable foundation of agricultural systems [7,8]. There is an urgent need for a technological revolution characterized by the deep integration of field agronomy and bioinformatics. In response, the concept of smart agriculture (Agriculture 5.0) has emerged, shifting the core paradigm from “experience-driven” to “data-driven” precision agriculture [2,9,10]. Plant multi-omics technology is likely to play a foundational role. By integrating multi-level data from genomics, transcriptomics, proteomics, metabolomics, epigenomics, and microbiomics, it can systematically decipher the complex patterns of plant life activities—from genetic blueprints to final phenotypic traits—and explore how plants respond to environmental changes [11,12]. This provides a new perspective for understanding and improving plants holistically. Therefore, in-depth research on the application and productization of plant multi-omics not only promotes a shift in plant biology toward a “systematic, dynamic, and networked” research paradigm [13,14,15], but also contributes to crop genetic improvement and the integration of microenvironmental adaptive ecological factors [16]. Additionally, it supports the construction of intelligent decision-support systems, offering key scientific and technological support for achieving high-yield, efficient, and sustainable agriculture [17].

**Figure 1:**
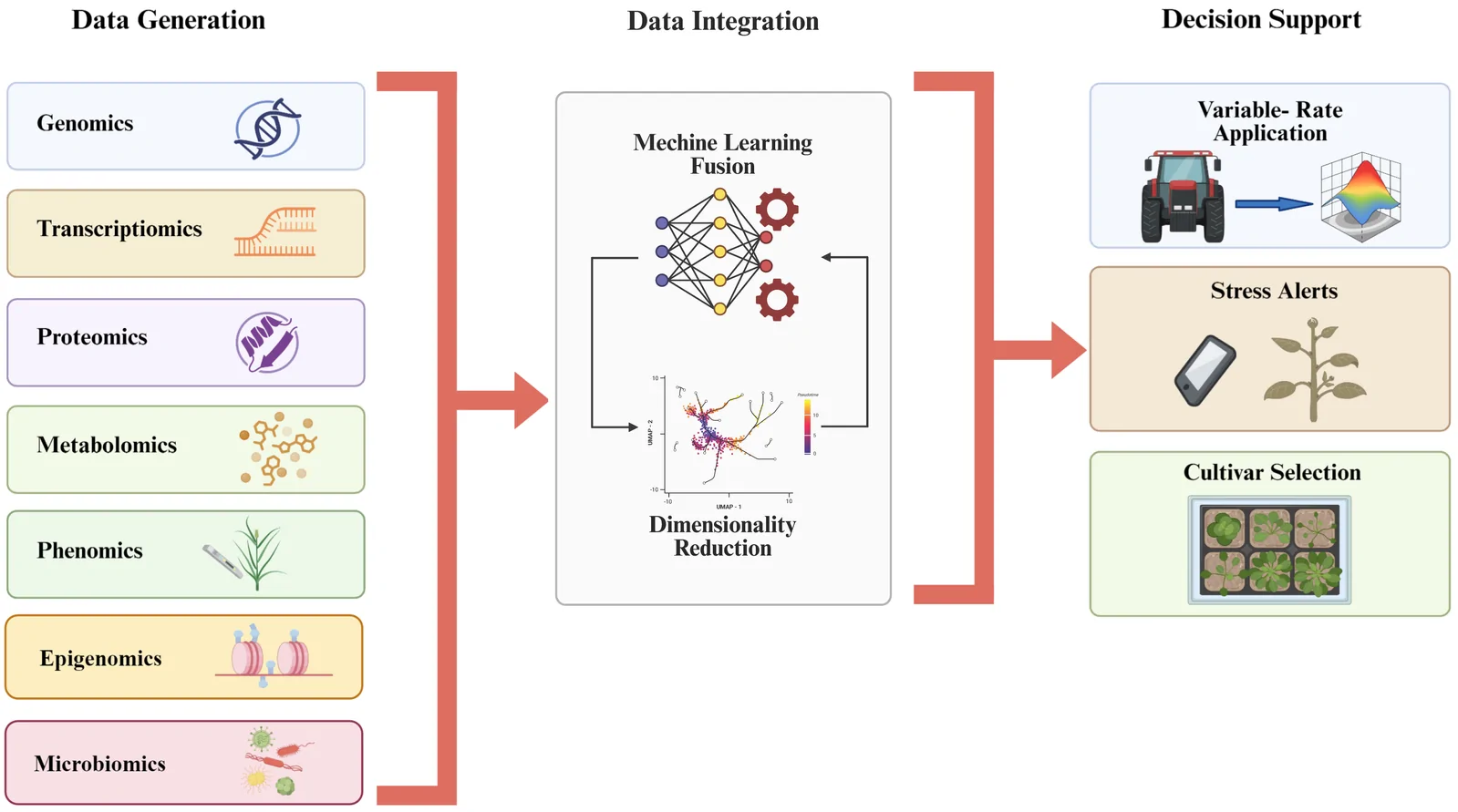
**Three-stage pipeline framework for multi-omics in precision agriculture.** In the data-generation stage, multi-omics data are collected across genomics, epigenomics, transcriptomics, proteomics, metabolomics, microbiomics, and phenomics. These heterogeneous layers are then integrated through machine-learning fusion and dimensionality reduction, and the resulting predictive models are translated into field-level decision support, including variable-rate input application, cultivar selection, and stress alerts.

While several recent reviews have surveyed individual omics layers or specific crops, comparatively few have adopted a translational perspective that traces plant multi-omics from mechanistic discovery to field-level application [11,13]. The novelty of this review lies in its “discovery-to-translation” framework, which combines an integrated approach to improving staple crops, remediating soil health and developing agricultural inputs. It also makes a clear distinction between precision breeding and precision agriculture and critically assesses reproducibility, molecular identification confidence and temporal dynamics. Many recent reviews either catalog omics technologies or concentrate on a single crop or application. This one looks across the main application areas through the same evidence lens, comments on how strong that evidence is, and relates each area to a realistic path toward translation, including the economic, regulatory, and data-governance constraints. Such an integrated and balanced treatment is uncommon in comparable reviews. The central premise of this review is that the greatest value of plant multi-omics in agriculture lies not in substituting for routine field measurements, but in discovering robust molecular markers, mechanistic rules, and biological targets that can subsequently be translated into scalable, low-cost diagnostic and breeding tools. Consequently, two discrete roles are distinguished. In precision breeding, multi-omics directly accelerates gene and marker discovery for crop genetic improvement. In precision agriculture, by contrast, multi-omics contributes more indirectly by informing the design of decision-support systems and input products. Concurrently, routine field-level decisions continue to rely on affordable proximal and remote sensing, Internet-of-Things (IoT) sensors, and phenotypic monitoring [9,10] (Figure 1). To develop this argument, the initial step is to outline the core multi-omics technologies and data-integration strategies. A critical evaluation of case studies is then conducted across three distinct application areas: namely, the genetic improvement of staple crops, soil health remediation, and omics-driven development of agricultural inputs such as biofertilizers and biostimulants. The strength of evidence is assessed in each case. Finally, we examine reproducibility, molecular-identification confidence, and temporal dynamics as cross-cutting limitations, and discuss future directions involving high-throughput phenomics, spatial omics, digital twins, and multimodal deep learning. Through this structure, we aim to move beyond a descriptive catalog of technologies and offer a balanced assessment of where plant multi-omics genuinely advances agricultural practice and where substantial gaps remain.

To keep the coverage transparent, the literature was retrieved from PubMed, Web of Science, and Scopus through combinations of terms such as ‘multi-omics’, ‘genomics’, ‘transcriptomics’, ‘proteomics’, ‘metabolomics’, ‘epigenomics’, ‘microbiome’, ‘single-cell’, ‘spatial transcriptomics’, ‘precision agriculture’, and ‘crop breeding’, with emphasis on work published between 2015 and 2026. Original research and authoritative reviews in plant science were given priority; non-plant (biomedical) material, purely descriptive single-layer studies, and non-peer-reviewed sources were set aside, and a case was retained only when it combined at least two omics layers or linked omics data to phenotypic or environmental measurements. This review is a qualitative, narrative synthesis rather than a systematic one, and the strength of the supporting evidence is appraised wherever a case is discussed.

## 2. Plant Multi-Omics Technologies and Methodological Framework

### 2.1. Plant Multi-Omics Technology System

Plant multi-omics is a high-throughput, multi-level biological research framework that systematically integrates the complete information flow from gene sequences to functional proteins, and ultimately to metabolic end-products [18]. Each “omics” technology within this framework is like a puzzle piece, collectively forming a comprehensive picture of biological phenomena. Genomics serves as the foundation of this system. Methods such as whole-genome sequencing (WGS) and reduced-representation sequencing delineate the complete genetic blueprint of an organism and identify DNA sequence variations, such as single nucleotide polymorphisms (SNPs) and structural variations (SVs) [19,20]. In contrast, transcriptomics focuses on the dynamic process of gene expression. Utilizing technologies such as RNA sequencing (RNA-seq), it measures the types and abundances of all RNA transcripts within a cell under specific conditions. This reveals when, where, and to what extent genes are activated or silenced [21]. Single-cell RNA sequencing (scRNA-seq) and spatial transcriptomics have markedly improved the resolution of transcriptomic analysis in recent years [22]. The two techniques are not interchangeable, however. Dissociated scRNA-seq isolates individual cells but loses their position in the tissue, and the protoplast isolation it requires can bias which cell types survive. Most spatial transcriptomics platforms keep spatial context but resolve spots rather than single cells, and subcellular platforms are still hard to apply in plants. The two are therefore often used together, so that cell-type-resolved expression can be placed back into its spatial context; recent syntheses of single-cell/nucleus and spatial maps of plant stress responses illustrate both the promise and the difficulty of these approaches in plants [23]. This advancement enables the detailed analysis of complex tissue and organ development processes. For instance, the construction of a single-cell multi-omics atlas in rice has significantly enhanced our understanding of cell differentiation and fate determination [24].

Proteomics investigates the expression, modification, and interaction of proteins, which are the primary executors of biological functions [25,26]. Employing mass spectrometry techniques, researchers can identify thousands of proteins and quantify changes in their abundance [27,28]. For instance, the high-throughput multi-omics atlas encompassing tens of thousands of proteins in wheat provides crucial proteomic-level evidence for understanding the functions of its complex hexaploid genome [29,30]. Metabolomics is concerned with the comprehensive and qualitative analysis of all small-molecule metabolites within an organism. As the direct products of the interaction between genotype and environment, metabolites reflect the physiological state of the cell [31,32,33]. Techniques such as liquid chromatography–mass spectrometry (LC-MS) enable researchers to understand how plants respond to external stresses and how their quality and nutritional traits develop [34]. Furthermore, epigenomics studies heritable changes in gene expression regulation that occur without altering the DNA sequence itself, primarily through mechanisms such as DNA methylation and histone modification. These modifications play a vital role in the way plants respond to environmental stress and in developmental regulation [35,36]. Finally, microbiomics broadens the scope of research to encompass not only the plant itself, but also its surrounding microbial communities, particularly those in the rhizosphere. Interactions between plants and microbes profoundly influence plant nutrient uptake, growth, stress resistance, and crop yield and quality [37,38,39,40] (Table 1).

### 2.2. Multi-Omics Data Integration Strategies

The power of multi-omics research lies in its integrative capability: using computational methods to combine data from different molecular levels in order to gain a deeper and more comprehensive understanding of biological systems. However, integrating diverse omics data, which vary in dimensionality, distribution characteristics, and noise levels, poses significant challenges [41]. Depending on the depth and complexity of the integration, multi-omics data integration strategies can be typically categorized into three hierarchical levels [13].

The first level is element-based integration (level 1). This is the most direct and commonly used method. Its core principle is to identify associations across omics layers at the level of individual molecular elements, such as genes, transcripts, or proteins. For example, calculating the correlation between gene expression (transcriptomics) and protein abundance (proteomics) can be used to screen for genes undergoing significant post-transcriptional regulation [13,42]. Similarly, integrating loci identified by genome-wide association studies (GWAS) with expression quantitative trait loci (eQTL) of nearby genes can substantially improve the precision of identifying causal genes. This method has the advantage of being intuitive and easy to interpret, but it ignores the intrinsic structure of biological pathways and regulatory networks [43,44,45].

The second level is pathway- or module-based integration (level 2). This method transcends the individual molecule, exploring instead the collective behavior of modules–groups of multiple molecules that collectively perform specific biological functions. For instance, researchers can analyze changes in key genes, transcripts, and metabolites within the same metabolic pathway (e.g., the phenylpropanoid pathway) across the genome, transcriptome, and metabolome respectively. This allows them to determine whether the pathway is activated or inhibited at a holistic level [46,47]. Weighted Gene Co-expression Network Analysis (WGCNA) is a typical representative method of this level. It clusters genes into distinct modules based on similarities in their expression patterns; each module represents a functionally related gene network. These modules can then be correlated with phenotypic correlation or metabolite enrichment, etc., to identify key regulatory modules that drive complex traits [48]. This strategy is closer to biological logic, but its outcomes are contingent on the precision of existing pathway annotations and module definitions.

The third and highest level is mathematical model-based integration (level 3). This is the most advanced and challenging approach, employing sophisticated computational models and machine learning algorithms to reveal implicit or non-linear relationships hidden within multi-omics data. Deep learning models, such as convolutional neural networks (CNN) and recurrent neural networks (RNN), can learn and predict the complicated agronomic traits directly from raw gene sequences, expression profiles, and metabolic profiles [49,50,51]. Multi-omics-assisted genomic selection (GS) models, such as genomic best linear unbiased prediction (GBLUP) and its extensions, construct a covariance matrix that simultaneously considers genomic and phenomic information to predict individual breeding values. These models have considerable potential for application in the context of breeding programs for crops such as wheat [52,53]. While such methods can reveal complex, cross-layer regulatory patterns that are overlooked by traditional approaches, their “black-box” nature poses significant difficulties for the biological interpretation of results.

### 2.3. Key Analytical Tools and Platforms

The explosive growth of multi-omics data has led to the development of a series of powerful bioinformatics analysis tools and platforms, which have significantly facilitated the transformation of data into biological insights. R and Python are the primary programming environments for data processing and integration. These offer classic packages for differential analysis, such as limma, DESeq2, and edgeR, as well as great machine learning libraries, including caret and scikit-learn [51,54,55]. In the field of network analysis, Cytoscape is a widely adopted platform for visual network exploration. It seamlessly interfaces with various molecular interaction databases (e.g., STRING, BioGRID) and analytical tools (e.g., MCODE, CytoHubba), and enables the visualization of gene regulatory networks (GRN) and protein-protein interaction networks (PPI) [56,57]. Regarding dedicated multi-omics platforms, specialized online analysis portals have significantly lowered the barrier for researchers utilizing multi-omics data. For example, the IPOP (Integrative Plant multi-Omics Platform) is an online tool specifically designed for cross-species comparison and evolutionary studies in plant multi-omics. It integrates multi-dimensional data (genomic, transcriptomic, epigenomic, etc.) from multiple species, and provides standardized analytical workflows. This allows users to conveniently perform tasks including conducting homologous gene analysis, evolutionary trajectory reconstruction, and trait association studies [58]. Another notable example is the WheatPro database, which was developed by a Chinese research team and is the world’s first high-throughput multi-omics atlas and database for wheat. It encompasses transcriptomic, proteomic, phosphoproteomic, and acetylomic data from 20 different tissues throughout the entire wheat life cycle. Through this platform, users can query gene expression across multiple dimensions, analyze co-expression networks and identify key genes related to agronomic traits. WheatPro provides unprecedented data resources and support for functional genomics research and molecular breeding in wheat [29]. Similar integrative resources have been established for other staple crops. In rice, single-cell multi-omics atlases couple transcriptomic and epigenomic profiles across tissues and developmental stages to provide cell-type-resolved regulatory maps for gene discovery [24]. In maize, dedicated multi-omics portals integrate genomic, transcriptomic, and metabolomic data to support trait analysis and predictive breeding [59].

## 3. Key Application Areas and Case Studies

### 3.1. Precision Agriculture and Decision Support Systems

The core objective of precision agriculture is to implement meticulous management of crops to maximize yield, optimize resource-use efficiency, and minimize environmental impact. Plant multi-omics technology provides comprehensive information from the molecular to macroscopic phenotypic levels, offering a robust scientific foundation for achieving this goal and serving as a key driver in constructing next-generation intelligent decision support systems (DSS) [18,60]. The traditional DSS is chiefly dependent on remote sensing data and elementary meteorological models. Conversely, the multi-omics-driven DSS is constituted by a sophisticated predictive system that integrates multi-dimensional information from genomics, metabolomics, microbiomics, and phenomics. The fundamental logic of the process involves the application of multi-omics technologies to facilitate a comprehensive analysis of the molecular response mechanisms and physiological characteristics of a specific variety (genotype) under particular environmental conditions [16,61,62]. This facilitates the establishment of sophisticated models that capture the interactions between genotype, environment, and phenotype (G × E × P) [52]. Subsequently, machine learning and deep learning algorithms-such as Random Forest, Support Vector Machine (SVM), and Convolutional Neural Networks (CNN)-are employed to train these high-dimensional multi-omics datasets. This process builds predictive models capable of accurately forecasting key indicators like crop growth, yield, stress resistance, and nutrient requirements [49,63]. For instance, a study was conducted on maize integrated SNP genotype data, high-throughput image-derived phenotypic traits (i-traits) from 16 developmental stages, and 133 primary metabolite data. With a Random Forest model, the reported accuracy of maize yield prediction rose from 0.32 to 0.43 [59]. This improvement came from a single study and a single population, so it is unclear whether it survives independent testing, holds across environments and years, or has real agronomic value. Nor is it established that adding these layers performs better than genomic selection alone.

In practice, such a DSS can be integrated with high-throughput phenotyping acquisition technologies such as drones, satellite remote sensing, and Internet of Things (IoT) sensors [16,52,64]. Drones equipped with multispectral or hyperspectral cameras can monitor real-time crop vigor, nitrogen levels, and water stress in fields. These dynamic phenotypic data are fed into the predictive models that have been trained using multi-omics data. Following rapid analysis, the models generate a series of “prescription maps”, providing farmers with precise information on which areas of a field require fertilization, irrigation, or pest/disease control, and the exact amounts needed [65]. The omics-derived inputs behind these decisions are specific: nitrogen-use-efficiency markers inform variable-rate nitrogen, stress- and disease-related molecules feed alerts and cultivar choice, and phenomic time series describe the current state of the crop. This can cut the waste and pollution that come with uniform, “one-size-fits-all” management and improve input-use efficiency, and in some cases yield and quality. Such gains, however, depend on the quality of the model and on field conditions; they are usually reported from prototypes or small trials and have rarely been measured at farm scale. Existing patented technologies showcase prototypes of such systems. For example, they use machine learning and remote sensing to analyze multispectral satellite images for crop classification and phenological monitoring to support agricultural decision-making [66,67].

It is worth clarifying how multi-omics connects to field-level prescription maps, since the two act at different points in the pipeline. Multi-omics works upstream: it identifies markers, candidate genes, and physiological parameters–such as a marker for nitrogen-use efficiency or a metabolite that signals stress–that indicate how a genotype is likely to respond to a given environment. The in-field decisions themselves are driven mainly by real-time sensing, weather, and soil data, which are compared against models that have been parameterized with those omics variables [10,65]. Omics thus defines what to predict, while sensors describe the current state, and a prescription map is the output of a sensor-driven model informed by omics rather than a direct omics readout. Few of the published prototypes spell out this division of labor, and worked examples showing which omics parameters enter a model—and how much they improve on remote-sensing-only baselines—are still rare.

For the G×E×P framework to be operational rather than conceptual, molecular layers need to be collected together with a defined set of environmental covariates—sowing date, temperature and rainfall series, soil texture, pH, and nutrient status—and with high-throughput phenotypes, ideally across coordinated multi-environment trials that span several sites and years [61,63]. In practice, most studies capture only a few of these variables, and often within a single environment, so the predictions they produce travel poorly.

A working multi-omics decision-support system can be broken down into a few concrete components. Its inputs are omics-derived predictors (for example, marker effects for nitrogen-use efficiency, resistance alleles, or stress-related metabolites), environmental covariates, and sensor time series; its outputs are a recommended cultivar, sowing date, or variable input rate. Regularized regression, random forest, and gradient-boosting models are the algorithms most often used, and uncertainty is better reported as prediction intervals than as a single number. Evaluation should rely on held-out environments and years, with prediction accuracy and, where possible, economic return as the metrics. Most systems so far have been tested only as prototypes or in small trials, and independent farm-scale validation remains uncommon. Digital twins that track a real field with omics-derived parameters are still conceptual in agriculture, and would need well-organized data, dependable mechanistic models, and substantial computing power before their day-to-day value could be judged [68,69].

### 3.2. Genetic Improvement of Major and Specialty Crops

Plant multi-omics technologies are profoundly transforming the paradigm for genetic improvement in the three staple crops: rice, wheat, and maize. By systematically deciphering the complex genetic networks that control yield, quality, and stress resistance, these technologies provide breeders with powerful, precise tools and abundant genetic resources (Table 2). In the examples below we separate four levels of evidence: candidate genes and related modules inferred from co-expression or association analyses; gains predicted within individual studies; mechanisms supported by functional experiments; and conclusions confirmed under field conditions.

**Rice (*Oryza sativa*)**: As the staple food for over half the global population, the genetic improvement of rice is crucial for ensuring food security [70]. The application of multi-omics technologies, particularly single-cell, has brought revolutionary breakthroughs in revealing the cell-specific regulatory mechanisms underlying rice development and stress responses. For instance, researchers utilized these technologies to simultaneously map chromatin accessibility and RNA expression profiles in over 116,000 rice cells across eight different tissues and organs [24,71,72]. This work not only meticulously reconstructed the gene regulatory networks that govern the differentiation and functional specialization of different rice cell types but also identified candidate cell-type-specific regulatory genes, including *RSR1* and *F3H* [24]. These genes came mainly from co-expression and regulatory-network analysis of the atlas, and their functions have not been confirmed by mutants, transgenic lines, or field trials; possible pleiotropic effects are also unknown. They are best regarded as candidate genes and potential regulatory nodes, rather than proven targets for crop improvement. These findings provide precise molecular targets for future directional improvement of rice organs such as roots, leaves, and panicles through gene editing or marker-assisted selection, thereby enhancing nutrient use efficiency and yield potential. Furthermore, integrated multi-omics analysis also plays a significant role in rice disease resistance research. For example, the gene *OsTPS1*, which encodes the enzyme responsible for synthesizing the sesquiterpene phytoalexin α-bisabolol, confers bacterial blight resistance in rice. Its natural variation in disease resistance was linked to promoter polymorphisms and the epigenetic regulation by JMJ705 through the methyl jasmonate pathway [73].

**Wheat (*Triticum aestivum*)**: Wheat is a typical allohexaploid with a complex and massive genome, which poses significant challenges to traditional genetics and genomics research. However, this complexity underscores the importance and value of integrated multi-omics analysis for wheat research [74,75]. Recently, Chinese research teams have achieved world-class breakthroughs in wheat multi-omics. They successfully constructed the first high-throughput multi-omics atlas (WheatPro), which includes transcriptomic, proteomic, phosphoproteomic, and acetylomic data from 20 different tissues across the entire life cycle. Based on this massive dataset, the researchers not only constructed a transcriptional regulatory network covering over 900,000 regulatory relationships but also discovered a novel protein module, *TaHDA9*–*TaP5CS1*. This module is associated with crown rot resistance in wheat, possibly through the regulation of proline accumulation. Because this evidence comes from co-expression and multi-omics association rather than direct functional assays, further validation is needed before it can be treated as a confirmed mechanism. This discovery not only reveals a new mechanism for wheat disease resistance but also provides a highly valuable gene target for molecular breeding of disease-resistant wheat [29]. Another study employed multi-omics to map the first high-resolution spatiotemporal profile of the wheat spike development. It systematically revealed the interactions between metabolites and gene expression networks, successfully identified key genes that regulate grain size, such as *TaOPR3* and *GL1*, whose superior allelic variations have been consistently selected and utilized in modern breeding [76]. These accomplishments fully demonstrate that multi-omics technologies help overcome the research challenges posed by the complex wheat genome and accelerate the discovery and utilization of its superior genes.

**Maize (*Zea mays*)**: An important crop for food, feed and bioenergy, the molecular mechanisms underlying its yield and heterosis are major research foci [77]. Multi-omics technologies provide powerful tools for dissecting these complicated issues. One study integrated genomic, metabolomic, and high-throughput phenomic data from maize recombinant inbred lines. Using machine learning algorithms for yield prediction, it found that integrating multi-omics data could predict yield more accurately than single genomic data alone. It also identified key regulatory pathways related to photosynthesis and grain development [59]. This indicates that, when it comes to predicting complex quantitative traits, incorporating metabolomic and phenomic information that reflect environmental responses can compensate for the limitations of additive effects determined solely by the genome [78,79]. Additionally, multi-omics analysis has also been used to compare the responses of different crops to environmental changes [16,80]. A meta-analysis focusing on rice, wheat, and maize found significant species-specific responses to climate warming in these three crops. For instance, moderate warming positively affected wheat biomass and yield; however, negative impacts emerged when the average temperature during the growing season exceeded specific thresholds (e.g., 13–15 °C). In contrast, maize yield components generally decline with increasing temperature [81,82]. These findings provide crucial scientific evidence for climate-smart breeding and cultivation management in different ecological regions.

Table 2 also lists two non-cereal crops, which show how far multi-omics applications extend. In tomato, combined transcriptomic and metabolomic analysis identified core transcripts and flavor-related metabolites under several low-intensity stresses, although most of this work was done under controlled or greenhouse conditions [83,84]. In tobacco, multi-omics profiling of lines carrying the carrot lycopene β-cyclase1 (*DcLCYB1*) gene showed coordinated up-regulation of abiotic-stress-related genes and proteins, matching the higher tolerance to high light, salt, and H_2_O_2_ and the greater biomass seen experimentally [85,86]. These cases indicate that multi-omics can uncover stress- and quality-related mechanisms beyond the major cereals, but whether the findings hold in field-grown crops under varying conditions is still unclear.

**Table 2:** Case studies of multi-omics applications in representative crops.

Crop	Omics Approach	Key Findings	Sample Size/Environments	Replication and Validation Status	References
Rice	Single-cell and spatial multi-omics with epigenetic profiling	Single-cell multi-omics atlas of >116,000 cells across eight tissues reconstructed cell-type-resolved regulatory networks; candidate cell-type-specific regulators (*RSR1*, *F3H*) proposed; *OsTPS1* confers bacterial blight resistance	>116,000 cells; 8 tissues; controlled (laboratory) conditions	Atlas-derived genes are candidates only (no mutant or field validation); *OsTPS1* functionally validated	[24,73]
Wheat	Integrated multi-omics (WheatPro atlas)	First high-throughput wheat multi-omics atlas (20 tissues); related module *TaHDA9*–*TaP5CS1* linked to crown rot resistance; *TaOPR3* and *GL1* associated with grain size	20 tissues; controlled (laboratory) conditions	Module inferred from co-expression/association, not functionally confirmed; *TaOPR3*/*GL1* variants selected in breeding	[29,76]
Maize	Genomics + metabolomics + phenomics with machine learning	Multi-omics prediction of yield exceeded genomic data alone; photosynthesis and grain-development pathways implicated	Recombinant inbred population; single study	Single-study predicted gain; independent and cross-environment validation pending	[59]
Tomato	Transcriptomics + metabolomics	Core transcripts and flavor-related metabolites identified under low-intensity stresses	Not reported; controlled/greenhouse	Controlled conditions; field validation pending	[83,84]
Tobacco	Transcriptomics + proteomics (DcLCYB1 lines)	Abiotic-stress genes and proteins up-regulated, consistent with greater tolerance to high light, salt, and H_2_O_2_ and with higher biomass	Not reported; controlled conditions	Effects confirmed experimentally under controlled conditions; field validation pending	[85,86]

### 3.3. Soil Improvement and Remediation

Soil health is the cornerstone of sustainable agriculture. Multi-omics technologies, particularly microbiomics and metabolomics, provide powerful tools for gaining a deep understanding of, and making improvements to, complex soil ecosystems. Traditionally, soil health has been assessed using physical and chemical indicators, such as organic matter content, nutrient levels, and pH. However, these indicators often fail to comprehensively reflect the most active and critical biological components and their functional states within the soil ecosystem comprehensively [87,88,89]. The introduction of multi-omics technologies allows us to re-examine soil health from a “biological activity” perspective. An integrated “physical–chemical–biological” framework is therefore emerging, in which multi-omics-derived microbial and metabolic profiles are interpreted together with conventional physicochemical indicators such as pH, organic matter content, and nutrient availability. This provides a more comprehensive and functional diagnosis of soil health [87,89].

Through high-throughput sequencing, microbiomics can comprehensively analyze the community structure and diversity of microbial communities (e.g., bacteria, fungi and archaea) and identify beneficial plant growth-promoting rhizobacteria (PGPR) or functional microbial groups that are critical for soil fertility. Beyond cataloguing community composition, the field is increasingly shifting from structural description toward functional prediction, using metagenomic and metatranscriptomic data to infer the metabolic and ecological functions that microbial communities perform in situ [38,90]. For example, research has found that root exudates from cover crops can significantly alter the functional trajectory of soil microbial communities. Specific components of those can selectively enrich particular microbial taxa that are capable of producing plant hormones (e.g., IAA, GA4) or participating in nitrogen cycling [90,91]. These findings provide a theoretical basis for agronomic measures that aim to directionally regulate the soil microbiome to enhance soil health and crop productivity, targeting the “rhizosphere exudate-microbiota” interaction. Metabolomics, on the other hand, can reveal biochemical processes in the soil at the metabolite level. The soil metabolome directly reflects biochemical processes including microbial activity, plant root exudation, and organic matter decomposition. Metabolomic analysis can be used to monitor the activity of biochemical pathways related to nutrient cycling, pollutant degradation and stress response [92]. A multi-omics study found that the impact mechanisms of biodegradable microplastics (PBAT-PLA) and conventional polyethylene (PE) microplastics had significantly different impacts on the crop rhizosphere environment. PE microplastics primarily affected root vitamin metabolism, whereas PBAT-PLA microplastics mainly interfered with lipid enrichment pathways, with the former having a relatively smaller effect [93]. This finding is useful for scientifically evaluating and rationally applying novel agricultural inputs, such as biodegradable plastic films. Additionally, multi-omics is used to evaluate the effectiveness of new soil amendments and remediation technologies. For instance, one study compared the efficacy of a plant-derived peptide bio-chelator fertilizer with that of a conventional synthetic EDTA chelated fertilizer under alkaline soil conditions. The study found that the bio-chelator fertilizer primarily alleviated stress by enhancing the phenylpropanoid pathway to support plant antioxidant defenses. This indicates its potential as a promising alternative in sustainable agriculture [34].

In summary, by revealing the molecular interaction networks within the soil–microbe–plant system, multi-omics technologies are advancing soil health management and remediation from largely empirical and descriptive approaches toward more mechanistic and precise understanding, particularly when omics-derived biological information is integrated with conventional physicochemical indicators.

The mechanistic link between root exudates, microbial function, and nutrient cycling becomes clearer once the specific exudate compounds, responding taxa, and mediating genes are identified. Flavonoids and strigolactones in root exudates, for instance, can recruit or activate particular rhizosphere taxa, which then express genes for auxin (e.g., IAA) or gibberellin biosynthesis, or for nitrogen fixation and nitrification, feeding back on plant hormone signaling and nutrient supply. Pairing exudate metabolomics with rhizosphere metagenomics and metatranscriptomics can help pin down these compound–taxon–gene links, but most evidence is still correlative and needs cultivation-based or genetic work to confirm causality. In the bio-chelate example, the increase in phenylpropanoid-pathway activity, seen as more flavonoids and lignin-related metabolites, is thought to strengthen antioxidant defense and cell walls and so ease alkaline-soil iron stress; the step-by-step link from pathway activation to improved tolerance has not, however, been fully traced in planta [34].

Soils contaminated with toxic elements are another growing area of application. Multi-omics can point to the plant and microbial genes that control the uptake, detoxification, and immobilization of elements such as cadmium, arsenic, and lead, and can guide approaches that combine gene editing, synthetic biology, and microbial stabilization. Recent work discusses how targets found through omics might be engineered into crops and microbial consortia for lasting soil detoxification–an issue that bears directly on food safety in contaminated regions and deserves more attention in the multi-omics and agriculture literature [94].

### 3.4. Omics-Driven Applications of Agricultural Products

Plant multi-omics technology is not only a powerful tool for fundamental scientific research but also acts as an engine driving innovation and commercialization in agricultural inputs, particularly in the development of biofertilizers and biostimulants [95]. These products utilize natural biological processes to improve nutrient utilization efficiency, enhance crop stress tolerance and ultimately boost crop yield. This represents a vital step towards achieving sustainable agriculture.

**Biofertilizers**: Chemical fertilizers are often inefficient in application and can cause environmental pollution. In contrast, biofertilizers introduce beneficial microorganisms to improve the plant rhizosphere environment or directly supply nutrients [96]. Multi-omics technology enables the molecular-level design of more efficient biofertilizers. Firstly, during strain screening, researchers can utilize metagenomics and transcriptomics to screen and isolate superior strains with specific functions (e.g., nitrogen fixation, phosphorus solubilization, potassium dissolution, or phytohormone production) from healthy plant rhizospheres or special habitats (e.g., saline-alkali land). For example, in a study on potatoes grown in alkaline loess conditions, through integrating transcriptome, metabolome, and rhizosphere microbiome, researchers found that broadcasting a biofertilizer containing *Bacillus amyloliquefaciens* EZ99 most effectively promoted potato growth, a mechanism potentially linked to the upregulation of genes in photosynthesis and sugar metabolism pathways [97]. Secondly, in product formulation optimization, multi-omics can help elucidate the synergistic effects of different application modes or additives. For instance, a study on the comprehensive utilization of *Camellia oleifera* by-products, guided by multi-omics analysis, developed a biofertilizer from root extracts through controlled fermentation. Field trials demonstrated a 18–24% increase in crop yield, while reducing chemical fertilizer application by 50%. This achieved two benefits: it was good for the environment and it saved money [98].

These examples are encouraging, but most biofertilizer studies are carried out under controlled conditions or in a single soil type, and reported gains such as the 18–24% increase with the Camellia oleifera by-product formulation may be specific to that environment and soil and may not repeat across sites or seasons. Field trials in a range of environments, with effect sizes and their variability reported clearly, are needed before broader conclusions can be drawn.

**Biostimulants**: Biostimulants are a category of non-nutritional substances that act by stimulating intrinsic physiological processes in plants. The result of this stimulation is an improvement in nutrient use efficiency, stress tolerance, and crop quality [99]. The intricacy of their mechanisms renders multi-omics an exceptional instrument for elucidating their functional mechanisms. Numerous studies using integrated transcriptomics and metabolomics have revealed the molecular-level action patterns of bio-stimulants from various sources, such as protein hydrolysates and seaweed extracts. For example, a study demonstrated that a protein hydrolysate (P) derived from *Gramineae* and its fraction with molecular weight below 1 kDa (F3) exhibit similar effects in alleviating salt stress in lettuce, yet they regulate distinct signaling pathways: F3 specifically interferes with the ethylene and MAPK signaling pathways, whereas P treatment primarily functions by upregulating genes associated with the synthesis of osmolytes and antioxidants [100]. Another study found that even under low nitrogen conditions, the same protein hydrolysate biostimulant could promote lettuce growth by upregulating genes and hormone levels involved in auxin and cytokinin synthesis. This indicates that its effects are closely related to plant physiological state and nutrient levels [101]. These detailed molecular mechanism studies not only deepen our understanding of plant physiology but, more importantly, provide scientific guidance for developing “on-demand,” more efficient, and more stable biostimulant products. In practice, knowing which component acts under which conditions is a first step towards more consistent formulations.

Two points should be kept in mind. First, these effects were measured under controlled or greenhouse conditions–F3 specifically interfered with ethylene and MAPK signaling, P mainly up-regulated osmoprotectant and antioxidant biosynthesis, and auxin- and cytokinin-related genes were up-regulated under low nitrogen–and effects measured this way may not carry over to field crops facing several stresses at once. Second, explanations built on a single pathway may oversimplify how these products work, so field trials in different environments, along with dose–response and reproducibility testing, are needed before the mechanisms can be turned into reliable products.

## 4. Challenges and Future Perspectives

### 4.1. Reliability and Biological Validity of Multi-Omics Measurements

As well as the computational challenge of integration, the reliability and biological validity of the underlying measurements merit meticulous scrutiny. Firstly, the concepts of reproducibility and transferability have yet to be thoroughly explored. It is imperative to differentiate between technical reproducibility (measurement consistency within a platform), biological reproducibility (consistency across biological replicates), and agronomic or environmental reproducibility (consistency across locations and seasons). It is evident that a significant proportion of multi-omics associations are reported from a limited number of environments or a single time point. Consequently, there is a necessity to assess the transferability of these associations to new genotypes, locations, and years. This can be achieved through multi-environment and multi-year validation, independent validation populations, and cross-validation schemes such as leave-one-environment-out or leave-one-year-out [12,80]. Secondly, analytical resolution and molecular-identification confidence differ markedly across omics layers. In untargeted metabolomics, for example, a detected LC-MS feature is not synonymous with an unambiguously identified metabolite; confidence levels should be distinguished among detected features, putatively annotated metabolites, MS/MS-supported annotations, and authentic-standard-confirmed metabolites [34,92]. Analogous uncertainty exists in the fields of transcriptomics, proteomics, microbiomics, and single-cell or spatial omics. Each layer of analysis is subject to its own inherent error, and thus integration does not automatically result in increased reliability. It is imperative that quality control, confidence scoring, orthogonal validation, targeted assays, and functional experiments are therefore essential. Thirdly, temporal dynamics and hierarchical disconnection must be considered when interpreting multi-omics datasets. Changes in mRNA abundance do not necessarily translate into changes in protein abundance or phenotype, and a single time point represents only a transient snapshot. As different omics layers operate on different timescales, integrating layers collected at the same time point may not capture causal sequences. Time-resolved multi-omics, the explicit modeling of cross-omics temporal lags, distinguishing between transient and sustained responses, and functional validation are therefore required. Notably, transcriptional pathway enrichment should not be directly equated with metabolic pathway activation in the absence of evidence at the level of proteins, enzyme activity or metabolites [42,46,47].

### 4.2. Computational and Translational Challenges in Multi-Omics Integration

Although plant multi-omics technologies have demonstrated considerable potential, there are still numerous formidable technical, biological and ethical challenges to overcome in order to translate them from the laboratory to the field. Firstly, challenges pertaining to data-level remain prominent. Multi-omics data are characterized by high heterogeneity, high dimensionality, and significant noise. The effective integration of complex data from diverse sources and scales to construct predictive models that are both accurate and biologically meaningful is a core bottleneck in current research [13]. A significant number of prevailing integration methodologies are characterized by a “black-box” nature. The complexity and lack of interpretability of these models result in a limited understanding of the underlying biological mechanisms, which hinders their credibility and application in breeding practice [52,102]. Secondly, almost all important agronomic traits are complex quantitative traits, which are jointly controlled by quantitative trait loci (QTLs) and environmental factors, with intricate genotype–environment (G×E) interactions [101]. This indicates that an outstanding genotype in one environment may exhibit substandard performance in another, thereby introducing a significant degree of uncertainty with regard to cross-environmental, transferable breeding. Capturing and deciphering genes, environments, phenotypes, and their intricate nonlinear relationships within a single model represent a huge challenge in computational biology. Finally, a gap persists between novel basic science and technology and their practical applications. There is a stark efficiency disparity between high-throughput, automated laboratory multi-omics analysis and time-consuming, labor-intensive, manual field phenotyping-a phenomenon known as the “phenomics bottleneck” [103]. Furthermore, the process of translating multi-omics research findings into actual productivity in the fields involves breeders’ adoption, seed production and promotion, and the promotion of omics products, as well as raising farmers’ awareness and acceptance.

Several practical pitfalls are common in multi-omics prediction. Batch effects from separate sample preparation, instrument runs, or platforms can create spurious associations, especially in transcriptomics, metabolomics, and proteomics, and are usually handled by randomization, blocking, and batch-aware normalization. Missing values are widespread in metabolomics and proteomics and need careful imputation rather than simple substitution, while the time lags and mismatches between layers make causal claims difficult. Small sample sizes, overfitting, and data leakage—for instance, selecting features before cross-validation—tend to inflate reported accuracy, so evaluation on independent test sets or through nested cross-validation, with uncertainty reported, is preferable; models that keep some interpretability are also easier to apply [41,51]. Data interoperability is a further requirement: shared metadata standards, controlled vocabularies, and reproducible workflows are needed before datasets can be reused and compared across studies [102]. A clear hierarchy of evidence, from single-study correlations to replicated and causally validated findings, is still often missing.

The translational gap also has an economic and institutional side that is seldom quantified. There is little public information on what multi-omics actually costs per sample or per experiment, yet cost largely decides which layers can enter routine breeding [102]. Genotyping, and to a lesser extent low-cost targeted metabolomics, are the layers most often used in breeding programs today, while untargeted proteomics and single-cell or spatial methods are still mostly confined to discovery. Other obstacles include the lack of agreed data standards, poor model transfer between environments, limited computing and phenotyping infrastructure, and the practical questions of farmer adoption, regulation, and business models. Regulation is itself a hurdle: approval rules for gene-edited or omics-derived varieties and for microbial products differ between countries and can hold up translation even when the science is ready.

Data governance and the reporting of negative results have received little attention. Datasets that follow FAIR principles, carry clear licenses, and are deposited in public repositories would be far more reusable; at present, ownership, intellectual property, and benefit-sharing are rarely settled in advance, and this matters most for smallholder farmers and for researchers in low- and middle-income countries, who often contribute samples but see little return. The picture is further distorted because unsuccessful or non-reproducible studies are seldom published, so the literature overstates how well the methods work. Reporting null and negative findings openly, together with the data and code behind them, would give a more honest account of what multi-omics can and cannot do.

Despite these challenges, the future development of plant multi-omics presents clear and exciting directions. Firstly, there will be a deep integration of technologies and methods. Future research will increasingly rely on empowerment by artificial intelligence (AI), particularly multi-modal deep learning models such as graph neural networks (GNNs) and transformer architectures. These models can handle the graph structures and sequence dependencies in multi-omics data more effectively, enabling deeper and more precise data fusion and phenotype prediction [41]. Secondly, comprehensive expansion in the spatiotemporal dimension. With the maturation and popularization of single-cell multi-omics and spatial transcriptomics technologies, future research will be able to decipher the spatiotemporal dynamic expression patterns of gene regulation within tissues, organs, and entire plant individuals at unprecedented resolution [35]. Aligning these high-resolution molecular maps precisely with high-throughput spatiotemporal phenomics data will enable us to understand plant growth, development and response processes dynamically and systematically, as if watching a movie. Thirdly, highly efficient and cost-effective new products will be created. Currently, agricultural composite microbial agents designed using multi-omics technology demonstrate significantly superior effects in improving soil microenvironments compared to single-agent formulations [104]. This objective is realized through the precise screening of combinations of functionally complementary strains. The application of these strains to soil or crop root systems has been demonstrated to result in more pronounced increases in soil enzyme activity and organic matter content. Furthermore, these agents also demonstrate greater effectiveness in alleviating the challenges of continuous cropping and in cleaning up pollution [105,106]. Fourthly, the construction and implementation of a digital omics system. The ultimate vision is to establish a complete, closed-loop analytical workflow spanning from genes to phenotypes and from phenotypes to cultivated environment management. This requires tightly integrating multi-omics data with high-throughput field or greenhouse phenomics platforms, such as drones, robots, IoT sensors, etc. Simultaneously, precisely simulating and predicting the operational states of real-world environments within virtual spaces enables unlimited “what-if” analysis in digital domains. This facilitates the identification of optimal management strategies that can then be applied to the physical world, achieving ultimate efficiency and sustainability in agricultural production [16,52,107]. In summary, the ability to overcome the challenges and capitalize on the opportunities presented will define the trajectory of plant science and agricultural technologies over the next decade.

A prioritized roadmap is more useful than a list of technologies. The most pressing problem is reproducibility and validation across environments, which calls for community metadata standards, multi-environment trials, and open analysis pipelines. Next comes model generalization and interpretability, which need larger and more diverse datasets, batch-aware and causally informed modeling, and independent validation. Lower cost and inexpensive targeted assays would then open the way to wider use, while governance frameworks and simple decision tools for farmers would support scaling. Stage one (roughly the next two years) would be to agree community metadata standards, open analysis pipelines, and the multi-environment trials needed to test reproducibility; stage two (two to five years), to build larger and more diverse datasets and adopt batch-aware, causally informed models, judged by prediction gain on unseen environments rather than on training data; stage three (five to ten years), to lower routine assay costs and put governance frameworks and simple farmer-facing tools in place. Progress at each stage can be measured—for instance, by the number of independently validated markers, the gain in prediction on unseen environments, and the cost per sample of the relevant assay.

## 5. Conclusions

**Figure 2:**
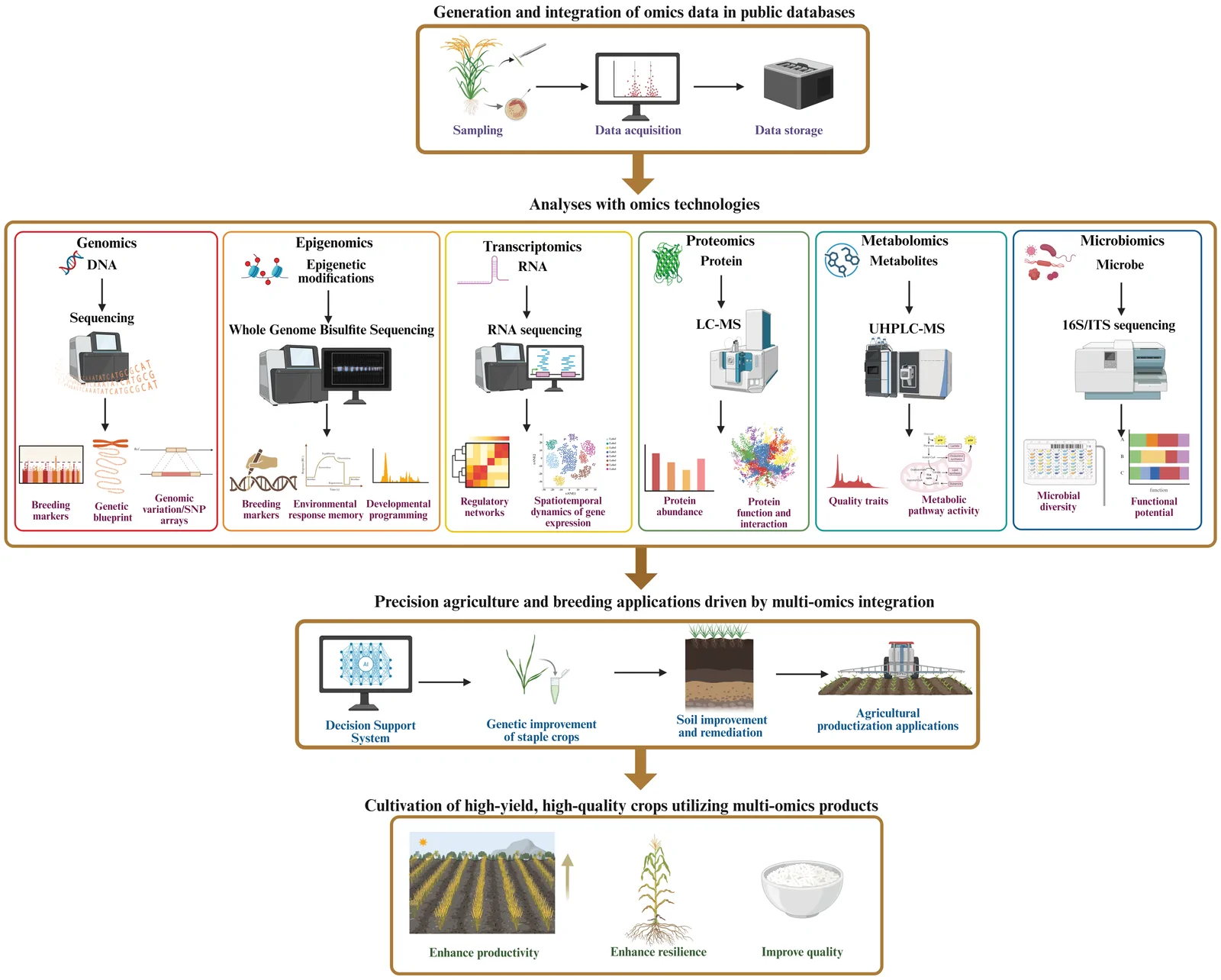
**Schematic flowchart of multi-omics integration driving precision crop breeding and agricultural applications.** The integrated application process of multi-omics technologies in crop breeding and agricultural production encompasses the complete technical pathway from sample collection, multi-omics data generation (including genomics, epigenomics, transcriptomics, proteomics, metabolomics, and microbiomics), data storage and integration, to final implementation in crop genetic improvement and precision agriculture. The figure emphasizes the role of multi-omics data in elucidating crop genetic variation, gene expression regulation, metabolic networks, and microbe–host interactions, as well as its practical applications. It also shows practical applications, such as using artificial intelligence and decision support systems for targeted breeding of high-yield, high-quality, and stress-resistant crops. Additionally, it includes soil improvement and remediation. Created in BioRender. Deng, F. (2026), https://BioRender.com/4bb9x6f.

This review has examined the application of plant multi-omics technologies in plants, spanning genomics, transcriptomics, proteomics, metabolomics, epigenomics, and microbiomics, in precision breeding, precision agriculture, soil health management, and the development of biofertilizers and biostimulants. The available evidence indicates that multi-omics integration is most mature in precision breeding. In this field, multi-omics integration has accelerated the discovery and pyramiding of superior alleles and the elucidation of complex trait networks in staple crops. In soil health and agricultural inputs, multi-omics has provided mechanistic explanations and efficacy-assessment tools, although these applications remain at an earlier stage of field validation (Figure 2).

At the same time, several limitations temper these advances. The reproducibility and transferability of many multi-omics findings across environments and years remain insufficiently tested, molecular identification is uncertain at several omics layers, and temporal disconnects between molecular levels complicate causal interpretation. These gaps mean that multi-omics is unlikely to become a routine field measurement in the near term; its greatest value instead lies in discovering robust markers and mechanistic rules that can be translated into affordable, scalable diagnostic and breeding tools. Realizing this potential will require multi-environment validation, community data standards, interpretable models, and closer integration with high-throughput phenomics, spatial omics, artificial intelligence, and digital twins.

In conclusion, plant multi-omics is a fast-moving field, but the evidence so far supports precision breeding more convincingly than day-to-day field management. Once limitations such as reproducibility, cross-environment validation, and data standards are addressed, multi-omics is poised to become a critical cornerstone for precision agriculture. Realizing that goal will depend on continued investment in basic research, data infrastructure, and translational validation.

## Figures and Tables

**Table 1:** Overview of the plant omics technology framework.

Omics Branch	Object of Study	Key Technologies	Core Information	References
Genomics	DNA sequence, gene structure, genomic variation	Whole-Genome Sequencing (WGS), Genotyping-by-Sequencing (GBS), SNP arrays	Genetic blueprint, variation sites, breeding markers	[19,20]
Transcriptomics	RNA transcripts (mRNA, non-coding RNA)	RNA sequencing (RNA-seq), Single-cell RNA-seq (scRNA-seq), Spatial Transcriptomics	Spatiotemporal dynamics of gene expression, regulatory networks	[21,24]
Proteomics	Proteins and their post-translational modifications (PTMs)	Liquid Chromatography–Mass Spectrometry (LC-MS), Data-Independent Acquisition (DIA)	Protein abundance, function, modifications, interactions	[29,30]
Metabolomics	Small molecule metabolites (<1500 Da)	Ultra-High Performance Liquid Chromatography–Mass Spectrometry (UHPLC-MS), Nuclear Magnetic Resonance (NMR)	Cellular physiological state, metabolic pathway activity, quality traits	[34]
Epigenomics	DNA methylation, histone modifications, chromatin accessibility	Whole-Genome Bisulfite Sequencing (WGBS), ATAC-seq, CUT&Tag	Gene expression regulation, environmental response memory, developmental programming	[35,36]
Microbiomics	Plant-associated microbial communities (bacteria, fungi, archaea)	16S/ITS rRNA gene amplicon sequencing, Metagenomics	Microbial diversity, functional potential, plant-microbe interactions	[38,40]

## Data Availability

No new data were created or analyzed in this study. Data sharing is not applicable to this article.
